# Supplementary material for: Content-rich biological network constructed by mining PubMed abstracts
Source: BMC Bioinformatics. 2004 Oct 8;5:147. doi: 10.1186/1471-2105-5-147 (PMC528731; doi:10.1186/1471-2105-5-147)
Supplement: Additional File 2 — The original results of the above study (non-essential files are deleted to keep the file size under the limit set by BMC bioinformatics). [file 1471-2105-5-147-S2.bz2 › chilibotAdditionalFile2/dip05/151ID8479754E546/html/CDC2_CCND1.html]

 


 **CDC2** and **CCND1** 
  
Found 821 abstracts in PubMed, retrieved 05.  
 

 What does Google say? 
 PDF only 
| .edu only 

---

**Interactive relationship** (e.g. stimulation, inhibition, etc)

**Non-interactive relationship** (e.g. studied together, co-existance, homology, etc.)

- In a series of 23 surgical biopsies of grade II and III oligodendroglioma, cyclin D1  [ **CCND1** ] , E, A, B1, CDK4 6, CDK2,  **Cdc2**  and p27 Kip.1 have been studied by immunohistochemistry and Western blot.  Ref: 12873740 Neurosci Lett, 2003
